# COVID-19 and dizziness: What do we know so far?

**DOI:** 10.1016/j.bjorl.2021.10.008

**Published:** 2022-02-28

**Authors:** Raquel Mezzalira

**Affiliations:** Universidade Estadual de Campinas (UNICAMP), Disciplina de Otorrinolaringologia Cabeça e Pescoço, Campinas, SP, Brazil

The SARS-CoV-2 virus is responsible for the current pandemic. Its main symptoms comprise fever, fatigue and dry cough, which can progress to dyspnea and respiratory failure. Other reported symptoms include headache, myalgia, odynophagia, nasal obstruction, rhinorrhea, anosmia, dysgeusia and gastrointestinal manifestations. On March 11, 2020, COVID-19 was declared as a global pandemic disease by the World Health Organization. Within weeks, the virus had spread to over 200 countries. In Brazil, the COVID-19 pandemic started on February 26, 2020, after the confirmation that a 61-year-old man from São Paulo who returned from Italy had tested positive for SARS-CoV-2, the cause of COVID-19. Since then and up to September 10, 2021, 20,974,850 cases have been confirmed, according to the Ministry of Health, causing 585,846 deaths. The number of people who have recovered from the disease to date is 20,016,161.^1^

It is not yet known whether SARS-CoV-2 can invade the neural pathways involved in the balance mechanisms, but initial observations imply this possibility. However, in most cases it is not possible to affirm whether the symptoms represent a worsening of a pre-existing disease, or a clinical manifestation that is fully virus-related, or a coincidental event.^2^

The incidence of dizziness in patients with COVID-19 ranges from 7 to 12% but studies need to be interpreted with caution due to the heterogeneity of the samples, data collection variation, low level of evidence, and lack of a control group.^3^, ^4^

It is clear that the isolation imposed by the pandemic has caused changes in people’s lifestyle, with emotional, somatosensory, metabolic, etc. consequences. The lack of social interaction leads to the onset and/or worsening of depression and anxiety, with a worsening in sleep quality. The practice of home office, often with inadequate facilities leads to postural vices, generating cervical pain and tension. Home confinement favors sedentary lifestyles and dietary mistakes, with abusive consumption of sweets causing weight gain. This situation has been called the “pandemic effect”, that, itself, may cause vertigo or other types of dizziness, regardless of vestibular involvement by the virus.^3^

Another fact to be taken into account is the vestibulotoxic effects of medications used in the treatment of COVID-19 infection, which remain unknown. Chloroquine and hydroxychloroquine are used at substantially higher doses than the usual ones and in a shorter period of time. Other frequently used medications include azithromycin, ivermectin, interferon and ribavirin. The possibility of being related to the onset or exacerbation of balance disorders should be considered. However, affirming that these symptoms are due to microthrombi or hypoxia, or due to the vestibulotoxic effects of COVID-19 therapy is not an easy task.^2^, ^4^

In the post infection period, some individuals develop fatigue, headache, and memory impairment. This situation has been called post-COVID syndrome. Patients who have been bedridden for a long period of time present with loss of muscle mass. In addition, the involvement of the central nervous system during the infection can trigger imbalance as a consequence, without changing the vestibular function.^3^

Therefore, it seems clear that there are several factors involved in the development of vertigo and other types of dizziness in this context of the ongoing pandemic, without necessarily compromising the vestibular system. However, some hypotheses are used to explain its damage during SARS-CoV2 infection. The first hypothesis suggests a direct involvement of the inner ear and vestibular nerve structures by the virus. To enter the cell, the SARS-CoV-2 virus depends on the angiotensin-2 converting enzyme receptor and the transmembrane serine protease 2. It has been demonstrated in rats that the receptors for these enzymes are present in the Eustachian tube, middle ear mucosal epithelium and inner ear. Thus, these receptors could act as a gateway for SARS-CoV-2 to enter the inner ear and affect the vestibular nerve, causing vertigo. On the other hand, some authors have defended the hypothesis of a potential inflammatory involvement of the inner ear vessels, eventually causing vasculitis or endothelitis. Balance disorders may be dependent on vascular damage, because inner ear structures are particularly susceptible to ischemia, due to their characteristics of terminal vasculature and high energy requirements. Other proposed mechanisms have been associated to the presence of a persistent inflammatory picture, with the production of proinflammatory cytokines that can impair inner ear functions, triggering an immune response mediated by the presence of the virus.^2^

Dizziness can manifest as an acute vertigo crisis with peripheral characteristics. The main differential diagnosis must be made with a posterior fossa stroke.^2^ Some patients have developed benign paroxysmal postural vertigo (BPPV) during the course of COVID-19 infection. Possible explanations would be a direct inflammatory action on the macula or the formation of microthrombi in the circulation, causing the degeneration and detachment of otoliths.^5^ In general, these clinical pictures are investigated and treated in the conventional way, without any particularities.

Whether the vestibular symptoms are a result of the “pandemic effect” or inner ear dysfunction due to the direct effect of the virus, vascular process, immune mediated reaction, or vestibulotoxicity, it remains unclear. Although the vestibular symptoms may appear during or after COVID-19 infection, it is not yet possible to affirm whether there is a cause-and-effect relationship or whether it is just a coincidence.

## Conflicts of interest

The authors declare no conflicts of interest.
